# A Rare Case of an Abdominal Aortic Thrombus Secondary to Syphilitic Aortitis

**DOI:** 10.7759/cureus.9397

**Published:** 2020-07-26

**Authors:** Raja Shekar Jadav, Tova Goldstein, Laith Alzyood, Mario Campana, Vitaly Adler

**Affiliations:** 1 Internal Medicine, St. Barnabas Hospital, New York, USA; 2 Cardiovascular Disease, Mayo Clinic, Rochester, USA; 3 Internal Medicine, Albert Einstein College of Medicine, Montefiore Medical Center, New York, USA

**Keywords:** aorta, syphilis, thrombus

## Abstract

Syphilis is a sexually transmitted spirochete infection whose presentation depends on the stage of infection. Currently, due to antibiotic treatment, tertiary syphilis is a rare clinical entity. When present, it is characterized by neurosyphilis, gummas, and cardiovascular infection. We present a case of a 64-year-old male who came with abdominal pain due to allergic colitis and was incidentally found to have a mural thrombus of his abdominal aorta. Following a negative workup and no etiologic cause of the thrombus, the patient was diagnosed with syphilitic aortitis. Previous cases have been seen in patients who present with infarction due to aortic thrombosis secondary to syphilitic aortitis. Practitioners must be aware that patients with tertiary syphilis, such as this patient, could have aortic thrombosis without any signs of ischemia and are at risk for infarction.

## Introduction

The clinical presentation of syphilis infection, caused by the spirochete Treponema pallidum, is dependent upon the stage of infection. Primary syphilis is characterized by a chancre that appears following exposure and resolves spontaneously. Following the dissemination of treponemal bacteria, secondary syphilis occurs and is manifested by a macular rash on the palms, soles, and face, typically three to six months following the appearance of the initial chancre. If left untreated, the infection can progress to tertiary syphilis, neurosyphilis, gummas, and cardiovascular infection. With the advent of antibiotics, however, the incidence of late-stage infections have decreased [1].

Cardiovascular syphilis manifests most commonly in the fourth to fifth decades of life, five to 40 years following primary infection. Aortitis, the hallmark of cardiovascular syphilis, occurs due to the invasion of the aortic wall by treponemal bacteria and the resultant endarteritis of the vasa vasorum and necrosis of the connective tissue and elastic fibers of the aortic media [2]. The ascending aorta is most commonly affected (50%) followed by the aortic arch (35%) and the descending aorta (15%) [3]. Few cases have been reported on the involvement of the abdominal aorta. Aortic aneurysms are the most common complication of aortitis, with 90% of cases occurring in the thoracic aorta and 10% in the abdominal aorta. Other complications include aortic regurgitation and coronary ostial stenosis [4].

Thrombosis of the aorta secondary to syphilitic aortitis is a rare but reported clinical occurrence, with thrombi occurring in the ascending and thoracic aortae. Here, we present a rare case of syphilis aortitis and its manifestation as a mural thrombus of the abdominal aorta in the absence of an aneurysm or evidence of atherosclerotic disease.

## Case presentation

**Figure 1:**
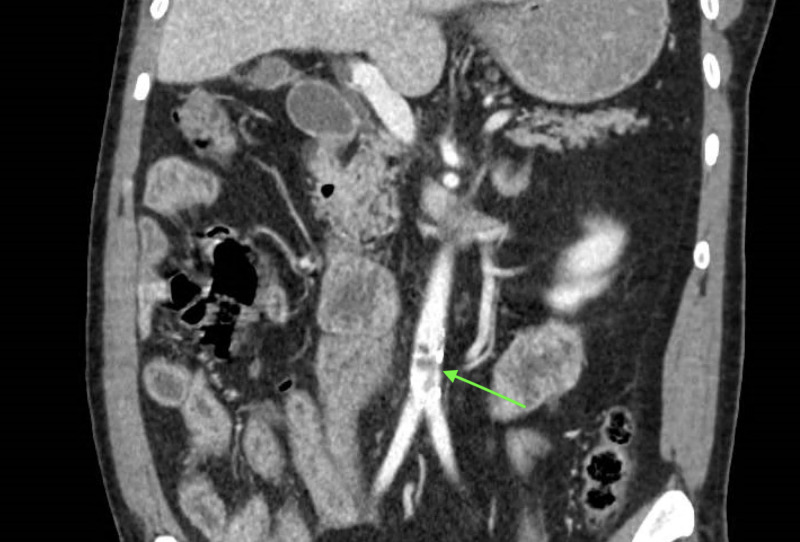
Abdominal CT scan with contrast Coronal view: showing irregular noncalcified thrombus in the distal infrarenal segment of the vessel significantly narrowing the effective lumen of the abdominal aorta CT: computed tomography

A 64-year-old male with a diffuse rash, abdominal pain, and non-bloody diarrhea for two days presented to the emergency department and was admitted for suspected allergy-related colitis following the ingestion of pineapple juice, a known allergy of his. Abdominal computed tomography (CT) with contrast was ordered and imaging revealed mesenteric edema, as well as the incidental finding of the abdominal aorta with a large amount of irregular noncalcified thrombus in the distal infrarenal segment of the vessel significantly narrowing the effective lumen (Figure 1).

No history of abdominal injury or trauma, leg claudication, impotence, rest pain, and tobacco use. Blood tests revealed leukocytosis 18,400 cells/microL, glycated hemoglobin (HbA1c) 7.1 %, and elevated C-reactive protein (CRP) 19 mg/L. Investigations for thrombophilic and autoimmune disorders, as well as cardiovascular disease and vasculitis, were negative. An echocardiogram revealed an ejection fraction of 70% without valvular disease and electrocardiogram, and Holter monitoring revealed normal sinus rhythm without premature ventricular contractions (PVCs) or episodes of ventricular tachycardia. Lower extremity ultrasound Doppler demonstrated normal triphasic flow without evidence of atheromatous changes, stenosis, or thrombosis. The aortic lesion in an otherwise healthy man without any other evidence of atherosclerotic disease led us to consider syphilis. Upon questioning, the patient remembered being diagnosed at one point but did not remember if he was treated, explaining that he has a history of penicillin allergy. Serology confirmed the diagnosis of syphilis with a reactive microhemagglutination assay for Treponema pallidum (MHA-TP), reactive rapid plasma reagin (RPR), and titer of 1:4. Aside from well-controlled diabetes, he did not have other cardiovascular risk factors. He was discharged on aspirin and statin therapy and follow-up with a surveillance duplex of the abdominal aorta every three months.

## Discussion

Mural thrombosis of the abdominal aorta in normal or minimally atherosclerotic vessels is a rare clinical entity. When present, thrombosis has been known to occur in the sac of an aneurysm due to dissection, saccular aneurysm, syphilitic aortitis, or secondary to malignancy, hematologic conditions, pelvic peritonitis, or abdominal radiation. While cases of thrombi due to syphilis have been recorded, to our knowledge, no cases have presented an abdominal mural thrombus in the absence of a concurrent aneurysm. In the absence of an aneurysm, arterial thrombi due to syphilis have been identified following workup for infarction and ischemic strokes. Documented cases include infarcts secondary to thrombosis of the ascending aorta [5] and aortic arch [6], as well as renal infarct secondary to thrombus in the thoracic aorta [7]. Our case is unique in that it was an incidental finding without evidence of embolic disease.

While both arterial and venous thrombi are composed of platelets and fibrin, arterial thrombi are typically found in areas where blood shear rates and blood flow are high while venous thrombi tend to occur at sites of healthy venous vasculature, where blood shear rates and flow are low. Due to these differences, antiplatelet agents tend to be more effective against arterial thrombosis while anticoagulant agents are better suited for venous thrombosis treatment. Risk factors for arterial thrombosis include tobacco smoking, elevated cholesterol, and hypertension, while venous thrombosis is associated with trauma, recent surgery, and malignancy. Arterial thrombus most commonly occurs in the arteries of the lower extremities secondary to pre-existing atherosclerotic plaque [8]. While our patient did have elevated low-density lipoprotein (LDL) cholesterol, he has been compliant with statin pharmacotherapy, does not smoke tobacco, and did not have signs of atherosclerotic disease. His lower extremity ultrasound Doppler demonstrated normal triphasic flow without evidence of stenosis or thrombosis, and his echocardiogram was within normal limits, without any valvular disease.

Given his lack of obvious risk factors for the development of arterial thrombosis, an alternative cause of syphilis infection was suspected. Approximately ⅓ of patients with untreated syphilis present with tertiary syphilis 10-30 years following initial exposure, and while rare, thrombosis of the aorta has been reported in syphilitic aortitis, a sequela of the tertiary disease [9]. Recent research studying Treponema pallidum has revealed that it is an invasive bacteria with the ability to readily cross-endothelial barriers through its ability to interact with, and activate platelets [10]. While further research is needed to expound upon these results and to elucidate the mechanisms behind mural thrombus formation in syphilitic aortitis, it aids in explaining the development of thrombosis in the absence of atherosclerosis.

## Conclusions

This case is important for practitioners, as it discusses a rare presentation of syphilitic aortitis. Unlike previously reported cases of aortic thrombus, which became evident secondary to infarction, this patient's thrombus was an incidental finding. It is possible that patients with untreated syphilis have similar thrombosis and are at risk for infarction, stroke, and possible death.
